# Supplementary material for: Cost-effectiveness results comparing heat-stable carbetocin & other uterotonics in postpartum heamorrhage prevention in Uganda
Source: PLOS Glob Public Health. 2025 Apr 22;5(4):e0003562. doi: 10.1371/journal.pgph.0003562 (PMC12013952; doi:10.1371/journal.pgph.0003562)
Supplement: Attachment — Submitted filename: Point_by_point response to the reviewers.docx [file pgph.0003562.s003.docx]

| **Querry** | **Response** |
| --- | --- |
| 1. Review the sentence that starts with however on line number200, it sounds incomplete | Thank you.  Rephrase it to read; “However, issues regarding the quality of oxytocin influence how physicians prescribe this medication” |
| 2. Line numbers 337 and 338 "When there was no CIs available, we assumed a standard error of 20%" What is the basis of spoor choice | Thank you for that comment.  The choice of 20% was assumed in the context that decision-making was not critically a high-stake one. |
| 3. I was not aware that HSC was proven superior to Oxy, as is implied by the first two rows of Table 2. I thought the RCT evidence showed “non-inferiority”.[1] In light of this, I think a much more extensive justification of Table 2 is required. | Thank you.  According to the RCT CHAMPION trial, HSC was non-inferior to oxytocin. However, a Cochrane network meta-analysis (U-NMA) by Gallos et al (Dec 2018), that included integration of the CHAMPION trial results, found that combinations of oxytocin with misoprostol or ergometrine, and stand-alone carbetocin, showed the highest efficacy. However, the combination regimens had more side effects, while carbetocin had none compared to oxytocin. The U-NMA concluded with “Carbetocin may be more effective than oxytocin for some outcomes without an increase in side effects.” We have modified the text to include that details. (page 11) |
| 2. Line 257-259: what is the basis for the utilization rates you have assumed for Oxy, Miso, and combination? | Thank you for raising this concern. The basis for the assumed utilization rates for Oxy, Miso and the Oxy+Miso combination were guided by Uganda’s Essential Maternal and Newborn Clinical Care Guidelines (May 2022), which identified these uterotonics as the preferred choices, and then specific input from several local Key Opinion Leaders determined the utilization percentage assumptions |
| 3. Line 259: I was not able to replicate the finding that overall risk of mortality due to PPH is 0.41%. Please elaborate on how you have derived this key quantity. | In the model, the overall risk of death is directly influenced by several factors, including the severity of the PPH event, type of healthcare centre used (primary, secondary or tertiary), the maternal age and need for referral to another type of healthcare centre; risk of death is indirectly influenced by the intervention based on its efficacy against PPH events. Baseline data on the risk of death by severity of PPH events and by the contributing factors are often not available in LMICs, including Uganda. Instead, the model utilizes the Excel Solver ([http://www.solver.com](http://www.solver.com/)) to calibrate the overall risk of mortality. In doing so, baseline risks for mortality are derived, stratified by PPH event severity, healthcare centre type, maternal age and need for referral.  The model calculated the overall risk of death due to a PPH event by entering  the maternal mortality ratio (MMR per 100,000 women) and the percentage of deaths due to PPH. Multiplying these two inputs provides the estimated number of PPH deaths per 100K women. This is then divided by the estimated number of PPH events per 100K women, determined within the model based on the treatment efficacy (for oxy, miso, and oxy+miso) and their respective market share from the first year in the budget impact model. For Uganda, the MMR is 189 (UDHS 2022), with 34% of the deaths due to PPH (estimate from Ugandan Maternal Death Reviews, 2022). Based on the assumed market share, the resulting overall risk of mortality due to PPH is 0.41% |
| 4. I did not find the probability of hemorrhage when patients received both Oxy and Miso. Did I miss it? Consider adding to Table 2. | Thank you for raising this omission.  We have included in the manuscript (see Table 2) the PPH probability percentages, as included in the model per the U-NMA, for the Oxy+Miso combination |
| [1] Widmer M, Piaggio G, Abdel‐Aleem H, Carroli G, Chong Y, Coomarasamy A, et al. Room temperature stable carbetocin for the prevention of postpartum haemorrhage during the third stage of labour in women delivering vaginally: study protocol for a randomized controlled trial. Trials 2016;17:143. | This statement is not clear to us and would love to understand what the question is. |
